# Hydro-Electro Hybrid Priming Promotes Carrot (*Daucus carota* L.) Seed Germination by Activating Lipid Utilization and Respiratory Metabolism

**DOI:** 10.3390/ijms222011090

**Published:** 2021-10-14

**Authors:** Shuo Zhao, Daniel Garcia, Yinglei Zhao, Danfeng Huang

**Affiliations:** 1 School of Agriculture & Biology, Shanghai Jiao Tong University, Shanghai 200240, China; zhaoshuo@sjtu.edu.cn (S.Z.); danielgarciafic@hotmail.com (D.G.); 2 College of Biosystems Engineering and Food Science, Zhejiang University, Hangzhou 310058, China; jjsshaka@zju.edu.cn

**Keywords:** carrot, hydro-electro hybrid priming, seed germination, lipid utilization, respiratory metabolism

## Abstract

Carrot (*Daucus carota* L.) is widely cultivated as one of the most important root crops, and developing an effective presowing treatment method can promote the development of modern mechanized precision sowing. In the present study, a novel seed priming technology, named hydro-electro hybrid priming (HEHP), was used to promote the germination of carrot seeds. Seed germination experiments showed that HEHP was able to increase the germination index (GI) and vigor index (VI) by 3.1-fold and 6.8-fold, respectively, and the effect was significantly superior to that of hydro-priming (HYD) and electrostatic field treatment (EF). The consumption and utilization rate of seed storage reserves were also greatly improved. Meanwhile, both glyoxysomes and mitochondria were found to appear ahead of time in the endosperm cells of HEHP through observations of the subcellular structure of the endosperm. Activities of isocitrate lyase (ICL), NAD-dependent malate dehydrogenase (MDH), pyruvate kinase (PK), and alcohol dehydrogenase (ADH) were significantly increased by HEHP. From transcriptome results, Kyoto Encyclopedia of Genes and Genomes (KEGG) pathways related to the glyoxylate cycle, glycolysis, gluconeogenesis, and the citrate cycle were significantly enriched and real-time quantitative PCR (qRT-PCR) analysis confirmed the expression pattern of 15 critical differentially expressed genes (DEGs) in these pathways. All DEGs encoding MDH, phosphoenolpyruvate carboxykinase (PEPCK), and PK were upregulated in HEHP; thus, it is reasonable to infer that the transformation of malate, oxalacetate, phosphoenolpyruvate, and pyruvate in the cytoplasm may be pivotal for the energy supply during early germination. The results suggest that the optimal effect of HEHP is achieved by initiating stored lipid utilization and respiratory metabolism pathways related to germination.

## 1. Introduction

Carrot (*Daucus carota* L.) is one of the most important root crops, and it is famous for its rich β-carotene and plant fiber contents [1,2,3]. According to the statistics of the Food and Agriculture Organization of the United Nations (FAO), China accounts for approximately 37% of the global carrot cultivation area and generates 48% of the global carrot yield, making it the largest carrot-producing country.

Seed germination is the key developmental transition that determines when the plant life cycle begins [4]. However, the carrot seed coat is poorly water permeable and seed germination is slow and patchy, thus causing obstacles to large-scale cultivation [5]. In addition, to reduce the cost of carrot seeds, mechanized precision sowing has been widely used in carrot cultivation; however, this method has higher requirements for the germination performance of seeds [6]. Therefore, it is of great practical significance to improve the germination rate and uniformity of carrot seeds through certain presowing treatment techniques.

As an effective presowing treatment technique to improve seed vigor, seed priming enables seeds to reach the pregermination stage when the radicle is about to break through the seed coat, thus allowing the seeds to carry out the metabolic process related to germination in advance [7,8]. Studies on a variety of crops have demonstrated that seed priming improves the germination characteristics and promotes seedling growth [9,10,11]. Hydro-priming (HYD) is low-cost and does not involve chemical reagent intervention; thus, it will not cause chemical substances penetration and residue on the seeds [12,13]. However, the HYD treatment takes a relatively long time, which increases the risk that seeds will be infected by harmful microorganisms [14].

The application of a high-voltage electrostatic field (EF) in seed presowing treatment belongs to the research field of agrophysics. Studies on a variety of crops, such as rice [15], potato [16] and tomato [17], have shown that this method is able to improve seed vigor in a short time with a simple device and operation. More concern has been given to EF treatment due to its weak damage to the seeds and near absence of chemical residue [18]. Nonetheless, the biological effects of EF tend to weaken in a short period of time [19,20]. Moreover, a previous study indicated that the application of EF to hydrated seeds tended to be significantly more effective than to dried seeds [21]. Therefore, our group has combined EF and HYD to form a hydro-electro hybrid priming (HEHP) method to realize the advantages of the two single initiation modes [21].

Extensive studies have suggested that normal utilization of seed storage reserves and efficient energy supply are necessary for successful germination [22,23]. Carrot seeds are dicotyledonous and have an endosperm with high oil content (mainly in the form of triacylglycerol) [24], which is used as an energy source for germination [25]. Regardless of the kind of seed priming technology applied, germination metabolism is directly activated by controlled seed hydration. In the early stage of seed hydration, oxygen is absorbed and carbon dioxide is released, which indicates that respiration is activated to provide energy for subsequent seed germination [26,27]. From the beginning of imbibition to radicle protrusion, mitochondria transport enzymes of the citrate cycle and ATP complex to the endomembrane [28]. Energy produced by respiration is essential to repair the damage accumulated in the dry state and support embryo growth required for completion of germination [29]. However, the mitochondria of some imbibed seeds are not fully developed. It is suggested that the seeds may also provide energy for germination through anaerobic respiration or other pathways. When pyruvate in imbibed seeds is blocked, anaerobic respiration will be activated [30]. In summary, the transformation from lipid metabolism to respiratory metabolism is critical for successful germination. The utilization of triacylglycerol (TAG) hinges on the glyoxylate cycle during germination for conversion to respiratory metabolism [31]. The effect of seed priming on germination may be related to its influence on the glyoxylate cycle, cardinal glycometabolism pathways, and even anaerobic respiration during germination initiation.

Various seed priming techniques have been used to promote the germination of carrot seeds [32,33,34]. Nevertheless, the physiological process related to carrot seed germination induced by seed priming has rarely been reported and how the carrot seed transcriptome responds to seed priming remains unknown. In the present study, HEHP technology was attempted as a novel means for improving the vigor of carrot seeds, and the effect of HEHP treatment was compared with that of HYD and EF alone. Furthermore, through observations of the subcellular structure of endosperm, evaluations of the physiological characteristics, and analyses of the transcriptome, we focused on lipid utilization and respiratory metabolism related to the response of carrot seed germination to seed priming. This study set out with the objectives of investigating the metabolic pathway related to carrot seed germination induced by the priming treatments, and the reason for the optimal effect of HEHP among all treatments.

## 2. Results

### 2.1. Induction of Carrot Seed Germination by Different Treatments

**Figure 1:**
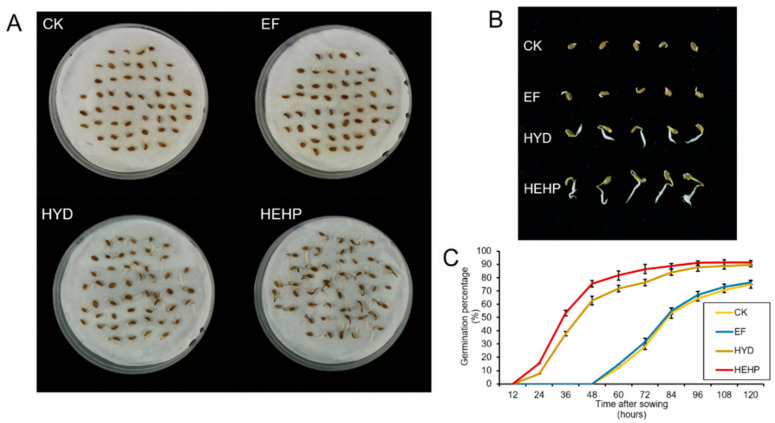
Seed germination and seedling growth status of carrot seeds after different priming treatments. (CK, the control; EF, high-voltage electrostatic field treatment; HYD, hydro-priming treatment; HEHP, hydro-electro hybrid priming treatment) (**A**) Photograph of seed germination experiment at 120 h after sowing; (**B**) Seedlings randomly selected from each treatment at 120 h after sowing; (**C**) Change trend of germination percentage with time after sowing.

Carrot seed germination was significantly promoted by HYD and HEHP, as shown in Table 1 and Figure 1. HEHP induced the highest germination potential (GP), germination rate (GR), radicle length (RL), fresh weight (FW), germination index (GI), vigor index (VI), and the shortest mean germination time (MGT). All the germination indicators of HEHP were significantly superior to those of the CK and EF treatments, indicating that the hybrid priming method had the optimum effect in promoting carrot seed germination. Compared to the control seeds, HEHP enhanced GP and GR by 220% and 22%, respectively, and GI and VI by 3.1-fold and 6.8-fold, respectively. EF significantly increased the FW, but the range of increase was less than that of HYD and HEHP and had no significant effect on the daily germination percentage. The RL, GI, VI, and MGT values were consistent with those of the control seeds. Meanwhile, HEHP tended to accelerate germination more than single HYD. Except for GR, the other germination indicators of HEHP were significantly superior to those of HYD (Table 1). The germination rate of the HEHP treatment remained significantly higher than that of the CK and EF treatments, and germination was observed only 24 h after sowing in HEHP and HYD, which was 36 h earlier than that of the CK and EF treatments (Figure 1C). At 120 h after sowing, the cotyledons of the HEHP-treated seedlings were almost exposed, while the radicles of the CK and EF treatments just extended out of the seed coat (Figure 1B).

### 2.2. Effects of Different Treatments on the Consumption and Utilization Rate of Seed Storage Reserves

**Figure 2:**
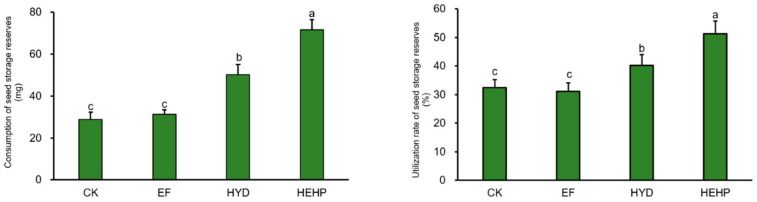
Changes of the consumption and utilization rate of carrot seed storage reserves after different priming treatments. Values represent the means ± SE from three biological replications. The different lowercase letters above the bars indicate significant differences in 95% probability level (*p* < 0.05, Duncan test was performed after ANOVA analysis).

As shown in Figure 2, HYD and HEHP significantly increased the utilization efficiency of seed storage reserves. HEHP led to the maximal consumption and utilization rate of seed storage reserves, and compared with control seeds, these parameters were enhanced by 150% and 58%, respectively, which showed that the storage reserve consumption speed of carrot seeds treated with HEHP was the fastest during germination. In addition, the consumption and utilization rates of HEHP were significantly higher than those of HYD. However, there were no significant differences in either metric between the EF and control seeds.

### 2.3. Effect of Different Treatments on Subcellular Structure of Endosperm

**Figure 3:**
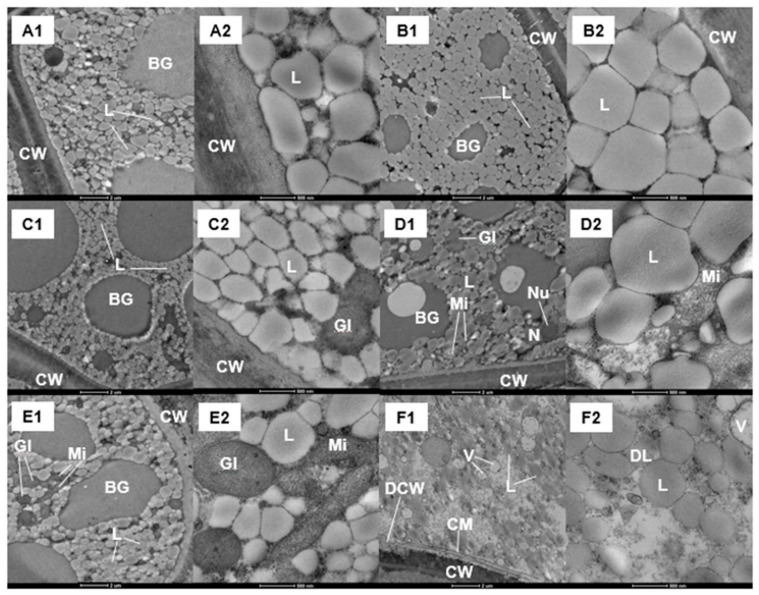
Changes of the subcellular structure of endosperm of carrot seeds after different priming treatments. (**A1**,**A2**) Two images from a CK seed endosperm before sowing; (**B1**,**B2**) Two images from a CK seed endosperm 20 h after sowing; (**C1**,**C2**) Two images from a HYD seed endosperm before sowing; (**D1**,**D2**) Two images from a HYD seed endosperm 20 h after sowing; (**E1**,**E2**) Two images from a HEHP seed endosperm before sowing; (**F1**,**F2**) Two images from a HEHP seed endosperm 20 h after sowing. (**A1**–**F1**) 4300×. (**A2**–**F2**) 22,000×. L: Oleosome; DL: Oleosome in the process of degradation; CW: Cell wall; DCW: Cell wall in the process of degradation; CM: Cell membrane; Mi: Mitochondrion; Gl: Glyoxysome; V: Vesicle; N: Nucleus; Nu: Nucleolus; BG: High electron dense substances.

The subcellular structure of the endosperm of carrot seeds from the different treatments was observed by transmission electron microscopy (TEM) before sowing (S0) and after 20 h imbibition (S20) (Figure 3). Since there was no significant difference observed between the CK and EF seeds, we have only shown the TEM results for the CK, HYD, and HEHP treatments. A large number of oleosomes were observed in the endosperm cells. For the CK seeds, the volume of oleosomes expanded, and the gap decreased after imbibition while the number did not change significantly, indicating that the degradation of stored lipids had not started. However, the number of oleosomes in the endosperm cells treated with HYD and HEHP declined after imbibition (Figure 3(D1,D2,F1,F2)). The number of oleosomes in the endosperm cells of seeds treated with HEHP decreased the most (Figure 3(F1,F2)), which showed the high speed of seed lipid degradation after imbibition. In addition, glyoxysomes and mitochondria were found in the HYD and HEHP treatments (Figure 3(C1,C2,D1,D2,E1,E2)), illustrating that lipid degradation and energy metabolism had been initiated in advance in carrot seeds after the priming treatment. Furthermore, degradation of the cell wall and oleosome, as well as a large number of vesicles, were found in endosperm cells of HEHP-treated seeds (Figure 3(F1,F2)).

### 2.4. Analysis of Key Enzyme Activities in the Glyoxylate Cycle and Respiratory Metabolism

**Figure 4:**
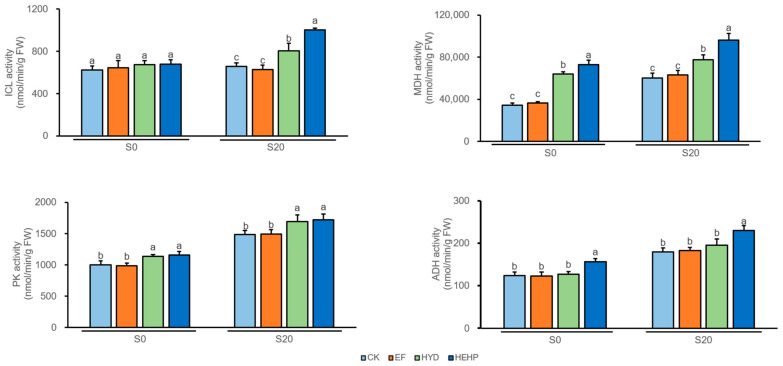
Changes of the key enzyme activities after different priming treatments. ICL, isocitrate lyase; MDH, NAD-dependent malate dehydrogenase; PK, pyruvate kinase; ADH, alcohol dehydrogenase. Values represent the means ± SE from three biological replications. The different lowercase letters above the bars indicate significant differences in 95% probability level (*p* < 0.05, Duncan test was performed after ANOVA analysis).

The activities of several key enzymes involved in the glyoxylate cycle and respiratory metabolism were further investigated, as shown in Figure 4. HYD and HEHP significantly increased the isocitrate lyase (ICL) activity in carrot seeds after imbibition (S20) while there was no significant change before sowing (S0). NAD-dependent malate dehydrogenase (MDH) activity was found to increase significantly by HYD and HEHP with the same trend at both S0 and S20, which was increased considerably by 112% before sowing. Both HYD and HEHP significantly improved the pyruvate kinase (PK) activity compared to the CK and EF treatments at both S0 and S20, although significant differences were not observed between HEHP and HYD. However, only HEHP significantly improved alcohol dehydrogenase (ADH) activity. Additionally, the key enzyme activities tended to increase at S20 compared to S0. In general, the activities of these enzymes in HEHP were significantly higher than those in HYD, except for PK activity at S20. HEHP was able to enhance the activities of ICL, MDH, PK, and ADH by 53%, 60%, 16%, and 28%, respectively, compared with the control seeds at S20.

### 2.5. Transcriptome Sequencing Data Statistics, Transcriptional Assembly and Functional Annotation

Based on the observation of the promotional effect of different presowing treatments on carrot seed germination, we used RNA-seq technology to sequence the transcriptome of carrot seeds at S0 and to reveal the metabolic pathway and key genes of carrot seeds activated before sowing by different processing methods. A transcriptome analysis of 12 samples was completed, which obtained 43.6~50.7 million raw reads and 43.1~50.2 million clean reads (98.76~99.08% of raw reads) from each sample. Then, the clean reads of each sample (35.9~43.7 million clean reads) were mapped to the designated carrot genomic database, and the alignment rate ranged from 81.47% to 88.05% (Table S1).

**Figure 5:**
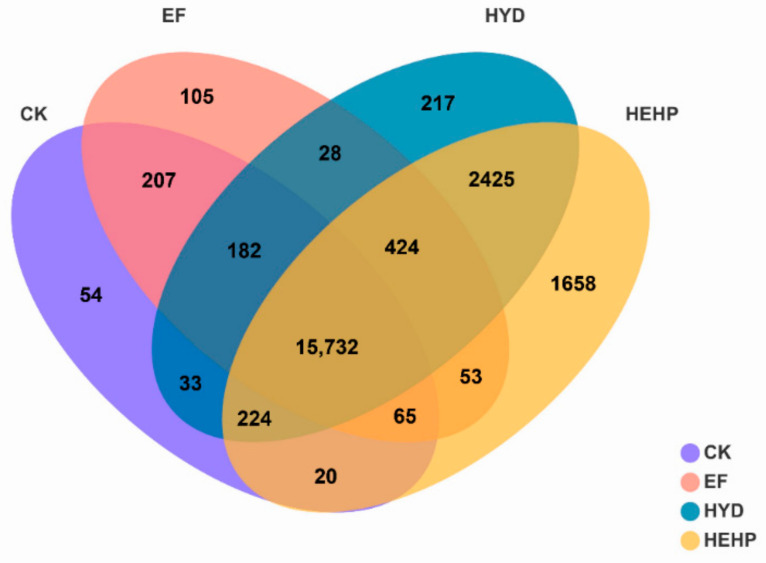
Venn diagram showing specific and overlapping identified genes among all the treatments.

Quantitative analysis of the gene expression level by the TPM quantitative index showed that 16,517, 16,796, 19,265, and 20,601 genes were identified in the CK, EF, HYD, and HEHP treatments, respectively. Among them, 54, 105, 217, and 1658 genes were specifically expressed in CK, EF, HYD, and HEHP, respectively, while 15,732 genes were coexpressed in all treatments (Figure 5). Both the total number of genes and the number of specifically expressed genes showed an increasing trend in CK, EF, HYD, and HEHP.

### 2.6. DEGs Statistics

As shown in Table 2, with the screening threshold of | log2FC | > 1 and *p*-adjust values ≤ 0.05, the maximum number of DEGs appeared between CK and HEHP (10,986 DEGs including 6655 upregulated and 4331 downregulated), while the minimum number of DEGs appeared between CK and EF (36 DEGs, including 31 upregulated and 5 downregulated). This result indicated that EF had little effect on the gene expression of carrot seeds with the DEGs between CK and EF accounting for only 0.3% of the total DEGs. In contrast, when the same EF was applied on the basis of HYD, namely HEHP, the gene expression of carrot seeds could be changed to a great extent. Thus, we focused on the DEGs among the CK, HYD, and HEHP treatments in the follow-up analysis.

### 2.7. Gene Ontology (GO) and Kyoto Encyclopedia of Genes and Genomes (KEGG) Enrichment Analysis of DEGs

**Figure 6:**
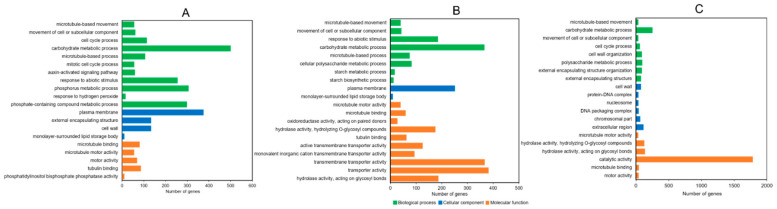
Gene ontology (GO) enrichment analysis of DEGs among CK, HYD and HEHP. (**A**) Top 20 enriched GO terms of the DEGs identified in CK vs. HEHP; (**B**) Top 20 enriched GO terms of the DEGs identified in CK vs. HYD; (**C**) Top 20 enriched GO terms of the DEGs identified in HYD vs. HEHP.

A GO enrichment analysis was performed on the DEGs among the CK, HYD, and HEHP treatments to reveal significant biological functions associated with the DEGs. The top 20 GO terms identified in the three groups (CK vs. HEHP, CK vs. HYD, and HYD vs. HEHP) are shown in Figure 6. Among these enriched GO terms, it is noteworthy that “carbohydrate metabolic process” appeared in each group and contained a large number of DEGs (501, 366, and 253 DEGs in CK vs. HEHP, CK vs. HYD, and HYD vs. HEHP, respectively), indicating that the priming treatment is likely to have a great impact on glycometabolism. The GO terms “movement of cell or subcellular component” and “microtubule-based movement” were also coenriched in all three groups. In addition, between the CK vs. HEHP and CK vs. HYD comparison, except for the GO terms mentioned above, “microtubule-based process” in the BP category, “plasma membrane” in the CC category and “tubulin binding” in the MF category were enriched in both groups. In the HYD vs. HEHP group, “microtubule-based movement” and “carbohydrate metabolic process” in the BP category, “external encapsulating structure” and “cell wall” in the CC category, and “microtubule motor activity” and “hydrolase activity (acting on glycosyl bonds and hydrolyzing O-glycosyl compounds)” in the MF category were the most enriched GO terms. Among the top 20 GO terms in this group, the maximum number of DEGs was enriched in the “catalytic activity” term (1786 DEGs), while the second largest number of DEGs was enriched in the “carbohydrate metabolic process” term (253 DEGs). These results suggested that EF may further activate a variety of hydrolase activities and promote cell substance transportation and cell division on the basis of HYD.

**Figure 7:**
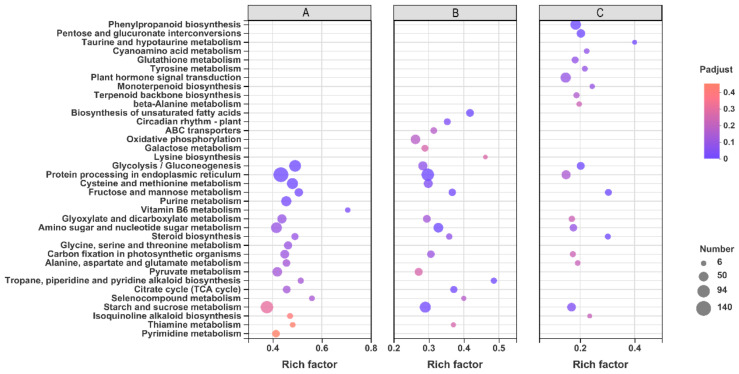
Kyoto Encyclopedia of Genes and Genomes (KEGG) pathway enrichment analysis of DEGs among CK, HYD, and HEHP. (**A**) The top 20 enriched KEGG pathways of the DEGs identified in CK vs. HEHP; (**B**) The top 20 enriched KEGG pathways of the DEGs identified in CK vs. HYD; (**C**) The top 20 enriched KEGG pathways of the DEGs identified in HYD vs. HEHP.

A KEGG enrichment analysis of the DEGs was conducted to investigate the induction of HYD and HEHP on the metabolic pathway of carrot seeds before sowing. The top 20 KEGG pathways enriched in DEGs of each group are shown in Figure 7. “Glycolysis/gluconeogenesis”, “glyoxylate and dicarboxylate metabolism”, “citrate cycle (TCA cycle)”, “starch and sucrose metabolism”, and “pyruvate metabolism” were in the top 20 enriched pathways in both the CK vs. HEHP and CK vs. HYD comparisons, and among them, “glycolysis/gluconeogenesis” was the most enriched pathway in CK vs. HEHP, indicating that respiratory metabolism-related pathways, such as glycolysis, the TCA cycle, and the glyoxylate cycle, were activated in the carrot seeds by the priming treatment. Meanwhile, “protein processing in endoplasmic reticulum”, “cysteine and methionine metabolism”, “fructose and mannose metabolism”, “amino sugar and nucleotide sugar metabolism”, and “steroid biosynthesis” were also in the top 20 of both groups. In the HYD vs. HEHP group, “glycolysis/gluconeogenesis”, “starch and sucrose metabolism”, and “glyoxylate and dicarboxylate metabolism” were also among the top 20 enriched pathways, reflecting that HEHP tended to activate these metabolic pathways to a greater extent than HYD. In addition to these coenriched pathways, “fructose and mannose metabolism”, “phenylpropanoid biosynthesis”, “pentose and glucuronate interconversions”, and “steroid biosynthesis” were also the top enriched pathways in the HYD vs. HEHP group.

### 2.8. DEGs Involved in the Glyoxylate Cycle and Respiratory Metabolism

**Figure 8:**
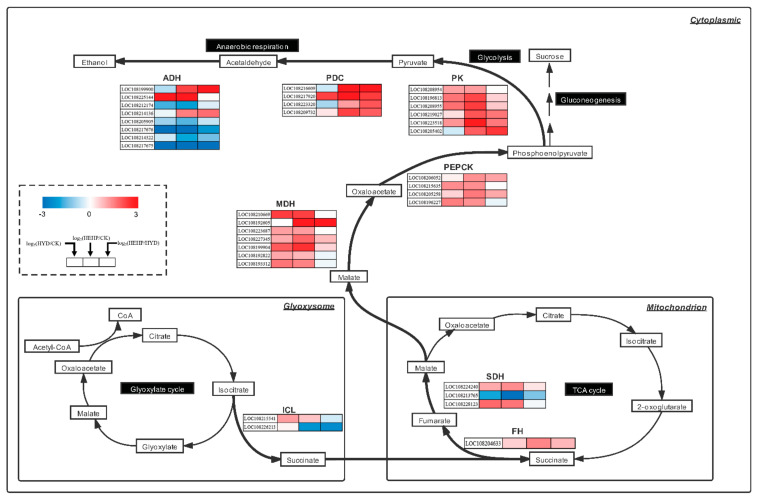
DEGs among CK, HYD and HEHP involved in the glyoxylate cycle and respiratory metabolism. ICL, isocitrate lyase; FH, fumarate hydratase; SDH, succinate dehydrogenase; MDH, NAD-malate dehydrogenase; PEPCK, phosphoenolpyruvate carboxykinase; PK, pyruvate kinase; PDC, pyruvate decarboxylase; ADH, alcohol dehydrogenase.

Based on the results of the GO and KEGG enrichment analyses, we focused on the DEGs encoding key enzymes of the glyoxylate cycle and related respiratory metabolic pathways. As shown in Figure 8 and Table S2, we identified a total of 35 DEGs involved in the above processes (none of these DEGs were in the CK vs. EF group). One of two DEGs (LOC108215541) encoding ICL was up-regulated in the HYD treatment compared with the CK. We also found that one DEG (LOC108204633) encoding succinate dehydrogenase (SDH) iron-sulfur subunit was upregulated in the HEHP treatment compared with the CK, while two DEGs (LOC108224240 and LOC108228123) encoding fumarate hydratase (FH) were upregulated in response to HYD and HEHP. All seven *MDHs* (LOC108210669, LOC108192605, LOC108223687, LOC108227345, LOC108199904, LOC108192822, and LOC108193312) were upregulated in HEHP or HYD compared with the CK, of which the expression of one *MDH* (LOC108192605) in HEHP was upregulated compared with HYD. The expression levels of all four DEGs (LOC108206052, LOC108215635, LOC108205258, and LOC108196227) encoding phosphoenolpyruvate carboxykinase (PEPCK) and six *PKs* (LOC108208954, LOC108196813, LOC108208955, LOC108219027, LOC108223518, and LOC108205402) were upregulated in HEHP or HYD, of which the expression of one *PEPCK* (LOC108205258) and three *PKs* (LOC108219027, LOC108223518, and LOC108205402) in HEHP were upregulated compared with HYD. It was also found that all four DEGs (LOC108216609, LOC108217920, LOC108223320, and LOC108209732) encoding pyruvate decarboxylase (PDC) were up-regulated in HEHP compared with the CK and HYD. However, of the eight *ADHs* identified, three genes (LOC108199900, LOC108225144, and LOC108214136) were upregulated by HEHP and HYD compared to the CK, of which two genes (LOC108199900 and LOC108214136) were upregulated by HEHP compared to HYD.

**Figure 9:**
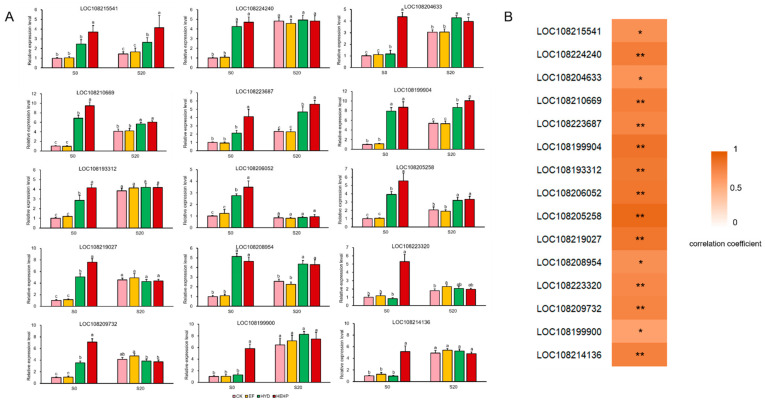
Real-time quantitative PCR (qRT-PCR) analysis of 15 selected DEGs at S0 and S20. (**A**) Expression patterns of 15 selected DEGs from qRT-PCR; (**B**) Correlation analysis between the results of qRT-PCR and transcriptome (Spearman correlation coefficient was used. * means significant at the level of *p* < 0.05. ** means significant at the level of *p* < 0.01). The corresponding relationship between gene ID and its encoded enzyme are as follows: LOC108215541, ICL; LOC108224240, SDH iron-sulfur subunit; LOC108204633, FH; LOC108210669, LOC108223687, LOC108199904 and LOC108193312, MDH; LOC108206052 and LOC108205258, PEPCK; LOC108219027 and LOC108208954, PK; LOC108223320 and LOC108209732, PDC; LOC108199900 and LOC108214136, ADH. The different lowercase letters above the bars indicate significant differences in 95% probability level (*p* < 0.05, Duncan test was per-formed after ANOVA analysis).

To verify the RNA-seq results, 15 DEGs were selected from the genes mentioned above for a real-time quantitative PCR (qRT-PCR) analysis of the seed samples at S0 and S20. The expression pattern of these DEGs at S0 obtained from qRT-PCR was positively correlated with the transcriptome results, reflecting that the transcriptome data were reliable (Figure 9B). Under the condition that the expression of some DEGs was upregulated by priming treatments at S0, the expression level of these genes became insignificant after imbibition at S20, such as DEGs encoding the SDH iron-sulfur subunit (LOC108224240), MDH (LOC108193312), PEPCK (LOC108206052), PK (LOC108219027), PDC (LOC108223320 and LOC108209732), and ADH (LOC108199900 and LOC108214136), while for other selected DEGs, the expression level of priming treatments after imbibition remained significantly higher than that of the CK (Figure 9A).

### 2.9. Analysis of DEGs Identified as Transcription Factors (TFs)

**Figure 10:**
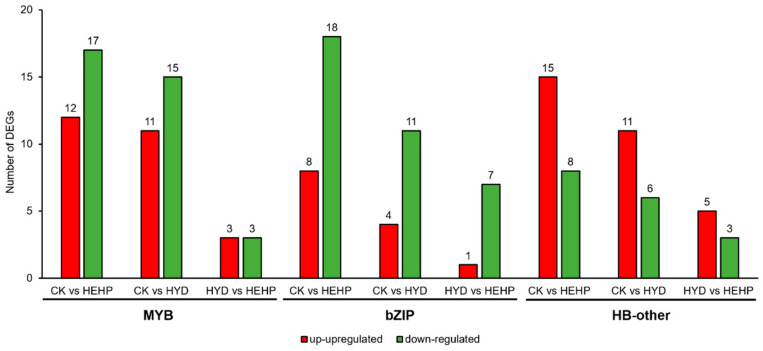
Statistics of DEGs among CK, HYD and HEHP encoding MYB, bZIP and HB-other family TFs.

A total of 355 DEGs were identified as TFs, which were distributed in multiple TF families (Table S3A). For the CK vs. HEHP, CK vs. HYD, and HYD vs. HEHP groups, 296 (83.7%), 204 (57.5%), and 78 (22.0%) DEGs were identified as TFs, respectively (Table S3B–D). MYB, bZIP, and HB-other family TFs accounted for the maximum number of DEGs among all three groups. In the CK vs. HEHP group, the MYB family had the maximum number of DEGs (29, 9.8%), followed by bZIP (28, 9.4%) and HB-other (24, 8.1%) (Table S3F). In the CK vs. HYD group, the MYB family was also the most abundant (29, 14.2%), followed by HB-other (18, 8.8%) and bZIP (16, 7.8%) (Table S3G). In the HYD vs. HEHP group, the bZIP and HB-other families had the maximum number of DEGs (9, 11.5%), followed by MYB (7, 9.0%) (Table S3H). As shown in Figure 10, 12 (41.4%), 11 (42.3%), and 3 (50.0%) MYB family TFs were upregulated in CK vs. HEHP, CK vs. HYD, and HYD vs. HEHP groups, respectively. Most DEGs encoding bZIP family TFs were downregulated by priming treatments, which accounted for 69.2% (CK vs. HEHP), 73.3% (CK vs. HYD) and 87.5% (HYD vs. HEHP). In contrast, HB-other family TFs were mainly upregulated by the priming treatments, which accounted for 65.2% (CK vs. HEHP), 64.7% (CK vs. HYD), and 62.5% (HYD vs. HEHP). In addition, most DEGs identified as HB-other family TFs encode homeobox-leucine zipper protein (HD-Zip) (Table S4G–I).

## 3. Discussion

Seed germination is a crucial factor for the propagation of plants, and it has also been the focus of widespread attention in agricultural production [35]. Rapid and uniform germination traits are urgently required for crop cultivation because they determine field performance and crop yield to a certain extent, particularly in the context of increasing uncertainty derived from climate change [36]. For carrots, especially with the rising cost of seeds, it is important to reduce the consumption and cost of seeds through mechanized precision sowing in cultivation, which has higher requirements for seed vigor. In addition, previous studies have noted that many species of the Apiaceae family, including carrots, have non-uniform and asynchronized seed germination, which may be a common problem in the Apiaceae family [37,38,39]. The current study on carrot germination and the response to seed priming will also contribute to extending our knowledge of the germination mechanism in the Apiaceae family and the conservation of Apiaceae germplasm resources. Here, we combine the EF treatment with conventional HYD technology and apply this method as a carrot seed presowing treatment for the first time. It should be pointed out that this method entirely relies on activating the vigor of the seed itself to promote germination with no external chemicals or growth regulators involved; therefore, it is environmentally friendly.

Our research showed that HEHP was able to increase the GI and VI of carrot seeds by 3.1-fold and 6.8-fold, respectively. It is somewhat surprising that this effect is not only better than that of the HYD treatment in this experiment but also better than the effect of earlier application on onion seeds [21]. Contrary to expectations, the EF parameter used in this study had no significant effect on most of the germination indexes, and the transcriptome results also corroborated this point. A possible explanation for this finding might be that the EF parameter used in the present experiment was widely selected by applying different EF parameters on the basis of HYD, but when this EF parameter was applied alone, it was found to have little effect. On the one hand, it shows that the combination of EF and HYD is capable of obtaining a more significant effect. Moreover, this result cannot negate the effect of using EF alone. Considering that many previous studies have proven the effect of EF utilization [40,41,42], it can be assumed that the optimum EF parameter is associated with the application form; that is, we conjecture that the best EF parameters are different in the case of HEHP and single use.

It is undeniable that seed germination requires abundant energy and carbon skeletons [8,23,43]. Seeds store oil in the form of TAG in oleosomes to meet the energy demand of heterotrophic-to-autotrophic conversion during seed germination and seedling formation [44,45]. The rate at which lipid reserves are utilized is likely to affect the establishment speed of seedlings, and accelerating the energy supply is a means to improve the germination rate [36]. The present transcriptome results also agreed with this viewpoint, with a tremendous number of DEGs enriched in numerous pathways related to lipid utilization and respiratory metabolism, such as “glyoxylate and dicarboxylate metabolism”, “glycolysis/gluconeogenesis”, “citrate cycle (TCA cycle)”, “starch and sucrose metabolism”, and “pyruvate metabolism”. Seed storage lipid cannot be used directly to provide energy for germination. The glyoxylate cycle, the TCA cycle, and gluconeogenesis are crucial for the transformation of stored lipids into soluble sugars for germination energy requirements [46]. ICL is one of the key enzymes in the glyoxylate cycle, which catalyzes the cleavage of isocitrate to glyoxylate and succinate, and succinate enters mitochondria for subsequent reactions [47]. MDH catalyzes the mutual transformation between malate and oxalacetate, which is involved in the glyoxylate cycle, the TCA cycle, Perl’s pathway, etc. [30]. PK and PEPCK are the key enzymes in glycolysis and gluconeogenesis, respectively, and also participate in Perl’s pathway [30,48,49]. Previous studies have pointed out that Perl’s pathway plays a crucial role in the rapid energy supply at the early stage of germination [30], which uses oxalacetate as a substrate to produce phosphoenolpyruvate under the action of PEPCK, and NADH under the action of MDH to produce ADP under the action of NADH-pyrophosphorylase. The products of these two reactions, phosphoenolpyruvate and ADP, were used as raw materials to produce pyruvate and ATP under the action of PK. Malate transported to the cytoplasm is either used for gluconeogenesis or as a substrate for respiration [50]. In addition, both in the Perl’s pathway and the last step of glycolysis, pyruvate produced by PK is mainly metabolized by anaerobic respiration in the early stage of germination. Acetaldehyde is produced under the catalysis of PDC, and ultimately ethanol is produced under the action of ADH to release ATP [51]. This pathway is also vital for the energy supply in the early stage of germination under anoxic conditions [52,53]. The application of HYD to rice seeds has been proven to enhance germination and seedling growth in anaerobic soils [54]. In our study, the consumption and utilization rate of seed storage reserves of HEHP were markedly higher than those of the other treatments. TEM observations of endosperm tissue before sowing and after imbibition also showed that the degradation and utilization rate of oleosomes in endosperm tissue was significantly increased after the priming treatment, especially after the HEHP treatment. Compared with the control, glyoxysomes and mitochondria were observed in the priming treatment. At the same time, the activities of ICL, MDH, PK, and ADH were significantly increased by HEHP treatment after 20 h of imbibition and the MDH, PK, and ADH activities treated with HEHP were significantly higher than that of the CK before sowing, which further confirmed the results of the TEM observations. One interesting finding is that the increase in MDH activity in HEHP was much greater than that of the other enzymes. This result is consistent with previous findings on watermelon seeds [50], which is perhaps associated with the multiple metabolic pathways in which MDHs are involved. Although HYD also increased enzyme activity; the promoting effect was less than that of HEHP. Hence, EF can be used as an effective supplement to HYD to expand the induction effect. These results indicate that the priming treatment is capable of inducing carrot seeds to activate the key enzymes of storage lipid utilization and respiratory metabolism in advance, thus raising the mobilization efficiency of storage materials and shortening the germination process.

In a further step, we investigated the effect of priming treatments on the above processes at the transcriptional level aimed at DEGs from transcriptome data. Existing research has found highly expressed *ICL*, *PDC*, and *ADH* genes during germination [55]. In our study, 35 DEGs in the priming treatments encoding ICL, SDH, MDH, FH, PEPCK, PK, PDC, and ADH were searched through the analysis of transcriptome data. It was found that all *PDCs* were upregulated in the HEHP treatment compared with the CK and even the HYD treatment, implying that anaerobic respiration might be an important channel of energy supply in the early stage of carrot germination. Additionally, in the early stage of germination, high levels of reactive oxygen species (ROS) generated from mitochondria can cause cell injury [56]; as a result, we speculate that maintaining a certain intensity of anaerobic respiration is conducive to avoiding this injury. One obvious finding was that all DEGs encoding MDH, PEPCK, and PK were upregulated in the HEHP treatment compared with the CK and even the HYD treatment. Based on this, we consider that the transformation of malate, oxalacetate, phosphoenolpyruvate, and pyruvate may be pivotal for the regulation of carrot germination, which belongs to both Perl’s pathway and part of glycolysis in the cytoplasm. It can also be regarded as a transition hub from lipid utilization to respiratory metabolism including anaerobic respiration. The priming treatments used in the present work might speed up the energy supply by promoting the efficiency of these reactions in the early stage of germination. In addition, the upregulation of some key genes provides evidence that priming treatments have prepared the key substances for seed germination during priming. We noticed that HYD did not upregulate the expression of some DEGs while HEHP upregulated the expression of these genes, such as part of the *MDH*, *PEPCK*, *PK*, *PDC*, and *ADH* genes, which is another possible explanation for the more remarkable effect of HEHP than HYD. The qRT-PCR analysis showed that some DEGs that were upregulated at S0 became insignificant at S20, although the enzyme activity analysis showed that the effect of the priming treatments on enzyme activity was mainly reflected after imbibition, which demonstrated that the effect of priming treatments was to promote the synthesis of key substances for germination before sowing. Thus, once the seeds were sowed, this advanced material preparation promoted the activation of related enzymes. In the meantime, the expression of related genes was no longer activated or only activated temporarily because this part of the process had been carried out during priming.

TFs are a fundamental element that regulates various abiotic stress responses as a molecular switch [57]. Seed priming imposes abiotic stress on seeds that represses radicle protrusion but stimulates moderate stress responses during hydration and desiccation [58,59]. Accordingly, the expression of TFs may be mediated in this process and participate in the regulation of germination-related metabolic pathways. Various TFs play a vital role in seed germination regulation [60,61]. A previous study pointed out that MYB family TFs contributed to regulating lipid utilization specifically in the embryo to ensure proper Arabidopsis seed germination [62]. Multiple studies have proven that MYB family TFs are essential in ABA signaling feedback regulation [63,64], which ensures that ABA is effective in inhibiting lipid catabolism during germination [62,65]. In addition, abscisic acid-insensitive proteins (ABIs) that belong to bZIP family TFs are directly involved in the regulation of the ABA signaling pathway [66]. ABI5 plays a positive role in the ABA signaling pathway in the inhibition of seed germination [61]. In the present work, the MYB family had a maximum number of DEGs in both the CK vs. HEHP and CK vs. HYD groups, and several ABI5 were identified as DEGs in the transcriptome data, indicating that the priming treatments likely regulated the expression of these TFs to control lipid utilization and regulate germination. The HB-other family is one of the most common TFs, among which HD-Zip is a unique transcription factor in plants [67]. In our study, many DEGs identified as HB-other family TFs were HD-Zip, and most of them were charactered as upregulated genes in HEHP. HD-Zip has been proven to be involved in regulating seed germination and hypocotyl elongation [68]. For instance, HD-Zip silencing delayed seed germination in Arabidopsis, and upon seed imbibition, increased GA levels release two HD-Zip TFs to activate lipase gene expression and start lipid utilization, thus enhancing germination potential [69]. Moreover, HD-Zip has been reported to contribute to a long hypocotyl phenotype [70,71]. It seems that HEHP may serve to synthetize HD-Zip TFs to promote lipid metabolism and hypocotyl elongation in early germination.

## 4. Materials and Methods

### 4.1. Plant Materials and Treatment Protocols

Carrot cultivar Naaisi was used in this study. The seeds were purchased from Shanghai Wells Seeds Co., Ltd. (Shanghai, China). The seed batch was harvested and processed in 2019 and stored under proper conditions (5 °C and 50% relative humidity).

For the HYD and HEHP treatments, the carrot seeds were first soaked in distilled water at 20 °C for 6 h. Then, for HEHP, the positive and negative electrodes of a BM-201 electrostatic field generator (made in Suzhou, Jiangsu, China) were connected to two 10 cm × 10 cm copper plates placed 1 cm apart, and then the seeds were placed on the lower plate (cathode) and exposed to a 2 kV/cm EF for 90 s after absorbing the surface moisture with absorbent paper. The EF parameters were widely selected by pre-experiments. Then, HYD and HEHP were imbibed in a climate chamber (QHX-300BSH-III, made in Shanghai, China) set at 22 °C at a 98% humidity for 48 h in the dark, and desiccated at 25 °C in a drum wind dryer until the initial weight was achieved. For EF, the seeds were only exposed to a 2 kV/cm EF for 90 s. The control (CK) was not treated in any way. The detailed treatment procedures are listed in Table 3.

### 4.2. Seed Germination Experiments

After removing the incomplete and damaged seeds, 50 carrot seeds were sown in a Petri dish covered with 3 layers of sterile filter paper and moistened with 4 mL of distilled water in a climate chamber (QHX-300BSH-III, made in Shanghai, China) set at 20 °C and 80% relative humidity. The photoperiod is set to 14/10 h of light/dark by using LED light (WEGA-18W, made in Qingzhou, Shandong, China) set at 40 µmol/(m^2^·s). Distilled water (0.5 mL) was added to the petri dish every 24 h after sowing. A seed was considered germinated when the radicle protruded through the seed coat. The number of germinated seeds and percentage of germination (%) every 12 h were recorded after sowing until day 5 after sowing. The germination percentage on day 3 and day 5 was defined as the *GP* and *GR*, respectively. The average *RL* and *FW* per 10 randomly selected seedlings were determined on day 5. The *GI*, *VI*, and *MGT* were calculated by the formulas presented below. Six biological replications were assessed for each treatment.

$$GI=\sum \frac{Gt}{Dt}$$

VI=GI×FW

$$MGT=\frac{\sum \left(Gt\times Dt\right)}{\sum Gt}$$

*Dt* is the number of days when the number of germinated seeds was counted; and *Gt* is the number of germinated seeds counted on the corresponding day.

### 4.3. Analysis of the Consumption and Utilization Rate of Seed Storage Reserves

Before sowing, 100 seeds were dried in a drum wind dryer at 104 °C for 20 h and then were weighed and recorded as the original dry weight (*W*0). One hundred germinated seedlings were taken from each treatment on day 5. The radicle and germ were separated from the remaining seeds, wrapped in tinfoil, and continuously dried in a drum wind dryer at 104 °C for 20 h. The dry weight of radicles and germs (*W*1) and the remaining dry weight of seeds (*W*2) were measured. The formula for calculating the consumption (*WS*) and utilization rate (*RS*) of seed storage reserves is as follows [72]. Three biological replications were assessed for each treatment.

WS=W0−W2

$$RS=\frac{W1}{W}$$

### 4.4. TEM Observation of Endosperm

The samples used for the TEM observation were conducted on the seeds from each treatment at S0 (before sowing) and S20 (during germination, 20 h imbibed after sowing just before HEHP began to germinate). The seed coat was cut open with a scalpel, and endosperm tissue with a length and width of approximately 1 mm × 1 mm was quickly cut in 2.5% glutaraldehyde on a glass slide. The endosperm tissue was soaked in 2.5% glutaraldehyde at 4 °C for 20 h and then washed with PBS for 4 times and 15 min each time. After soaking in 1% osmium tetroxide for 2 h, PBS was used to soak and rinse 3 times for 15 min each time. Then, they were dehydrated in 50% ethanol, 70% ethanol, 90% ethanol, a mixture of 90% ethanol and 90% acetone (*v*/*v* = 1:1), and 90% acetone for 20 min, and then soaked in 100% acetone 3 times for 20 min each time. The samples were immersed in a mixture of acetone and epoxy resin (*v*/*v* = 1:1) for 2 h, immersed in a mixture of acetone and epoxy resin (*v*/*v* = 1:2) overnight, immersed in pure resin for 6 h, and immersed in pure resin at 60 °C for 48 h before slicing with an ultrathin microtome (Leica EM UC7, Leica, Wetzlar, Germany). Finally, the subcellular structure of the endosperm was observed by TEM (Talo L120C G2, Talo, Houston, TX, USA). For each treatment, the endosperm tissues of 9 seeds were observed.

### 4.5. Determination of Key Enzyme Activities in the Glyoxylate Cycle and Respiratory Metabolism

The samples used for enzyme activity determination were conducted on the seeds (approximately 0.2 g for each sample) at S0 and S20. All samples were frozen in liquid nitrogen and stored in a refrigerator at −80 °C. The ICL (EC 4.1.3.1), MDH (EC 1.1.1.37), PK (EC 2.7.1.40), and ADH (EC 1.1.1.1) activities were determined by assay kits (Suzhou Comin Biotechnology Co., Ltd., Suzhou, China). The enzyme activities were calculated by monitoring the rate of decrease in the absorbance value at 340 nm which is the position of the NADH absorption peak. A total of 24 samples (4 treatments × 2 sampling time points × 3 biological replications) were used for the analysis of each enzyme.

### 4.6. RNA Extraction, Library Preparation and RNA Sequencing Analysis

The samples used for RNA sequencing analysis were collected from the seeds from each treatment before sowing. All samples were frozen in liquid nitrogen and stored in a refrigerator at −80 °C. A total of 12 samples (4 treatments × 3 biological replications) were used for analysis.

#### 4.6.1. Extraction of Total RNA

Total RNA was extracted from the seed samples using Plant RNA Purification Reagent, according the manufacturer’s instructions (Invitrogen, Waltham, MA, USA), and genomic DNA was removed using DNase I (TaKaRa, Shiga, Japan). The concentration and purity of RNA were detected by a NanoDrop2000 (Thermo Scientific, Waltham, MA, USA). The integrity of the RNA was detected by agarose gel electrophoresis, and the RIN value was determined by an Agilent 2100 Bioanalyzer System (Agilent, Santa Clara, CA, USA).

#### 4.6.2. Illumina Sequencing

A TruSeq^TM^ RNA sample preparation kit from Illumina (San Diego, CA, USA) was used to construct the library, and then the paired-end RNA-seq sequencing library was sequenced based on the Illumina NovaSeq 6000 sequencer (2 × 150 bp read length) platform by Shanghai Majorbio Bio-pharm Technology Corporation (Shanghai, China). The raw paired end reads were trimmed and quality controlled by SeqPrepb (https://github.com/jstjohn/SeqPrep) and Sickle (https://github.com/najoshi/sickle) with default parameters. Then, clean reads of each sample were mapped to the carrot reference genome (https://www.ncbi.nlm.nih.gov/genome/?term=Daucus carota).

#### 4.6.3. Gene Expression Statistics

Based on the results of the comparison to the genome and genome annotation file, the read counts of each sample gene were obtained by using RSEM (http://deweylab.biostat.wisc.edu/rsem/), and then the TPM (transcripts per million reads) method was used to calculate the expression level of each transcript.

#### 4.6.4. Screening and Analysis of Differentially Expressed Genes (DEGs)

Based on the quantitative expression results, the differentially expressed genes between the two groups were analyzed, and the DEGs between the two groups were obtained. The differential analysis software was performed using DESeq2 with | log_2_FC | > 1 and *p*-adjust values (corrected *p*-value) ≤ 0.05 which indicated DEGs. The software Goatools (https://github.com/tanghaibao/GOatools) and KOBAS (http://kobas.cbi.pku.edu.cn/home.do) were used for the GO and KEGG pathway enrichment analyses of the DEGs.

### 4.7. qRT-PCR Analysis

Total RNA was extracted from the seed samples at S0 and S20 according to the method mentioned in Section 2.5. The selected DEGs and primers designed with Primer 5.0 are listed in Table S5. The inner reference used the carrot gene *actin-7* (LOC108202619). Reverse transcription amplification was performed with a Goldenstar RT6 cDNA synthesis kit ver 2 (Tsingke Biotechnology Co., Ltd., Shanghai, China). qRT-PCR was performed with 2×T5 Fast qRT-PCR Mix (SYBR Green I) (Tsingke Biotech, Beijing, China) on a CFX Connect Real-Time PCR Detection System (Bio-Rad, Hercules, CA, USA). The qRT-PCR amplification parameters were as follows: 95 °C for 1 min; then 40 cycles of 95 °C for 15 s, 60 °C for 15 s, and 72 °C for 30 min; followed by 1 cycle of 95 °C for 5 s, 60 °C for 1 min, and 50 °C for 30 s. The relative quantitative results were calculated by the 2^−ΔΔCt^ method according to the Ct values. Three biological replications were performed for each reaction.

### 4.8. Statistical Analysis

Statistical analyses of the germination characteristics and physiological indicators were carried out based on an analysis of variance (ANOVA) with IBM SPSS Statistics 19. Duncan’s multiple range test was used to compare the mean values in different treatments. Two-tailed probability values (*p*-values) were considered significantly different.

## 5. Conclusions

Under the induction of the seed priming treatments used in the current study, the germination process of carrot seeds was shortened and the germination indexes were significantly improved. The application of the same electrostatic field on hydropriming, namely hydro-electro hybrid priming, i.e., one novel physical presowing treatment method, contributed to promoting the germination of carrot seeds to a greater extent, and the effect was better than that of HYD and EF alone. The subsequent determination of the consumption and utilization rate of seed storage reserves and the observation of subcellular structure of the endosperm demonstrated that the priming treatments, especially HEHP, promoted the utilization of storage lipids in carrot seeds. The key enzyme activities of the glyoxylate cycle, glycolysis, the citrate cycle, and anaerobic respiratory metabolism were significantly increased by the HEHP treatment. Furthermore, the above metabolic pathways were significantly enriched based on the transcriptome analysis, and the qRT-PCR analysis confirmed the expression pattern of critical DEGs in different processes involved in the enriched pathways. Various TFs were also identified from the transcriptome data as potential participants in lipid utilization and germination regulation. One significant finding of this study is that the transformation of malate, oxalacetate, phosphoenolpyruvate, and pyruvate in the cytoplasm may be pivotal for the regulation of carrot germination, and this process may extend from lipid utilization to respiratory metabolism. Taken together, the effect of HEHP is to improve the efficiency of the energy supply in the early stage of germination by initiating storage lipid utilization and respiratory metabolism pathways related to the germination of carrot seeds and then promoting germination.

## Figures and Tables

**Table 1:** Effects of different treatments on carrot seed germination characteristics.

Treatment	GP/%	GR/%	RL/mm	FW/g	GI	VI	MGT/d
CK	27.0 ± 2.4c	74.7 ± 3.0b	6.84 ± 0.40c	0.058 ± 0.004d	20.06 ± 0.48c	1.16 ± 0.09c	4.29 ± 0.02a
EF	29.3 ± 1.6c	76.3 ± 2.3b	7.47 ± 0.26c	0.062 ± 0.003c	20.90 ± 0.15c	1.29 ± 0.05c	4.28 ± 0.02a
HYD	76.3 ± 2.3b	89.7 ± 1.5a	17.01 ± 1.48b	0.120 ± 0.002b	51.98 ± 1.16b	6.23 ± 0.02b	3.59 ± 0.02b
HEHP	86.3 ± 2.0a	91.3 ± 1.6a	22.96 ± 1.09a	0.129 ± 0.003a	61.40 ± 1.85a	7.93 ± 0.35a	3.47 ± 0.03c

Values represent the means ± SE from six biological replications. The different lowercase letters in the same column indicate significant differences in 95% probability level (*p* < 0.05, Duncan test was performed after ANOVA analysis).

**Table 2:** Statistics of differentially expressed genes.

Group	Total DEGs	Up	Down
CK vs. EF	36	31	5
CK vs. HYD	6808	4234	2574
CK vs. HEHP	10,986	6655	4331
EF vs. HYD	6612	4053	2559
EF vs. HEHP	10,721	6531	4190
HYD vs. HEHP	3853	2940	913

**Table 3:** Treatment procedures.

Treatment	Soaking	Exposure in Electrostatic Field	Imbibition in Climate Chamber	Desiccation
CK	×	×	×	×
EF	×	√	×	×
HYD	√	×	√	√
HEHP	√	√	√	√

## Data Availability

The sequencing data presented in this study are available on request from the corresponding author.

## Supplementary Materials

The following are available online at https://www.mdpi.com/article/10.3390/ijms222011090/s1.
